# Short-Term Effects of Kinesio Taping and Cross Taping Application in the Treatment of Latent Upper Trapezius Trigger Points: A Prospective, Single-Blind, Randomized, Sham-Controlled Trial

**DOI:** 10.1155/2015/191925

**Published:** 2015-09-29

**Authors:** Tomasz Halski, Kuba Ptaszkowski, Lucyna Słupska, Małgorzata Paprocka-Borowicz, Robert Dymarek, Jakub Taradaj, Gabriela Bidzińska, Daniel Marczyński, Aleksandra Cynarska, Joanna Rosińczuk

**Affiliations:** ^1^Department of Physiotherapy, Opole Medical School, Katowicka 68, 45-060 Opole, Poland; ^2^Department of Obstetrics, Faculty of Health Science, Wroclaw Medical University, K. Bartla 5, 51-618 Wroclaw, Poland; ^3^Department of Clinical Biomechanics and Physiotherapy in Motor System Disorders, Faculty of Health Science, Wroclaw Medical University, Grunwaldzka 2, 50-355 Wroclaw, Poland; ^4^Department of Nervous System Diseases, Faculty of Health Science, Wroclaw Medical University, K. Bartla 5, 51-618 Wroclaw, Poland; ^5^Department of Physiotherapy Basics, Academy of Physical Education in Katowice, Mikolowska Street 72, Building B, 40-065 Katowice, Poland

## Abstract

Kinesio taping (KT) may be a new treatment in patients with myofascial trigger points (MTrPs). A new method available for taping practitioners is cross taping (CT). The main objective was to determine how CT, KT, and medical adhesive tape (sham group) affect the subjective assessment of resting bioelectrical activity and pain of the upper trapezius muscle (UT) in patients with MTrPs. 105 volunteers were recruited to participate. The primary outcome was resting bioelectrical activity of UT muscle as assessed by surface electromyography (sEMG) in each group and pain intensity on a visual analog scale (VAS). Assessments were collected before and after intervention and after the 24-hours follow-up. No significant differences were observed in bioelectrical activity of UT between pre-, post-, and follow-up results. In three groups patients had significantly lower pain VAS score after the intervention (CT—*p* < 0.001, KT—*p* < 0.001, and sham—*p* < 0.01). The Kruskal-Wallis ANOVA showed no significant differences in almost all measurements between groups. The application of all three types of tapes does not influence the resting bioelectrical activity of UT muscle and may not lead to a reduction in muscle tone in the case of MTrPs.

## 1. Introduction

Myofascial pain syndrome (MPS) is one of the most frequent causes of musculoskeletal problems [1–3]. Myofascial pain is defined as pain which comes from myofascial trigger points (MTrPs) in muscle that are considered as hyperirritable spot located within a taut band of skeletal muscle. MTrPs are characterized by local tenderness without referred pain and local tenderness with referred pain and also by muscle dysfunction (weakness, fatigue, stiffness, and poor blood flow), restricted range of motion (ROM), change of motor pattern, poor posture, and limited physical, professional, and social activity [3–9]. Two types of MTrPs are distinguished: latent and active. Latent MTrPs cause local and referred pain with palpation and active MTrPs cause pain at rest and on palpation (“spontaneous pain”) [1, 5–10].

Publications about the MTrPs diagnosis indicate that the upper trapezius (UT) is the muscle in which MTrPs occur very often [7, 9–14]. MTrPs within the UT may cause neck pain, chronic upper-quarter pain, headache, migraine, or shoulder pain [12–15].

There are many ways to treat myofascial pain. The treatment algorithms can contain noninvasive methods like educational programs, behavioral cognitive therapy, medication, and physical therapy or physiotherapy (spray and stretch, general exercises, myofascial release, massage, Jacobson's muscle relaxation, autogenic training, manual therapy, neuromuscular techniques, electrotherapy—ultrasound, interferential therapy, transcutaneous electrical nerve stimulation, pulsed shortwave therapy, and laser therapy) [14–23]. The invasive treatments for MTrPs include injections with dry needling, local anesthetics, corticosteroids, and botulin toxin [21, 22, 24–27].

More and more publications suggest that kinesio taping (KT) may be a new treatment option and indicate the possibility of the use of KT in patients with musculoskeletal problems [28–32], including MTrPs [32–36]. KT is a therapeutic taping technique developed by Dr. Kenzo Kase (Japan, 1979). This technique uses an elastic tape that is thin and more elastic than conventional bandages. The tape can be stretched to 140% of its original length and applied to the skin [28, 31, 34]. KT is used as an alternative to athletic taping to support the fascia, muscles, and joints. In addition, in the literature it can be found that KT can increase the ROM, reduce swelling, inflammation, and bruising, enhance blood circulation, enhance strength and muscle tone, or be used in muscle spasms and cramping prevention and to speed recovery of overused muscles [32–34, 37–42]. Most of the research is related to the use of KT in relieving pain, specifically reducing pain and disability in patients with chronic, nonspecific back pain [29]. It seems that KT can also be used to combat pain in patients with MTrPs [38].

A new form of tapes available for taping practitioners is cross tapes. Cross tapes are small, polyester tapes with an adhesive acrylic coating. The water-resistant cross tapes are free of medication and active ingredients and mostly can be used for local points of pain, trigger or acupuncture points, tense muscles, painful joints, headaches, or painful scars. The tapes are applied directly over points of pain. Depending on the wearing conditions, they can adhere to the skin for a period up to several days. The tapes are available in different sizes (M, L, and XL) and, in contrast to KT, they are not elastic and are unable to follow the skin when it is stretched. Most information about cross tapes is on manufacturer's website; however, to our knowledge, there are no good quality research studies that evaluate the effectiveness of using cross taping (CT) [43]. Therefore, the influence of CT on body structures and functions needs to be confirmed by objective research.

The lack of the strongest types of research (meta-analysis, systematic reviews, or randomized controlled trials) on MTrPs treatment using CT and the lack of clear methodology of cross tapes application prompted the authors to perform an evaluation of effectiveness of CT. The main objective was to determine how CT, KT, and medical adhesive tape (sham group) affect the subjective assessment of pain and resting bioelectrical activity of the UT muscle in patients with MTrPs. A secondary objective was to evaluate cervical ROM before and after the intervention. Additionally, a comparison of the results was conducted between CT, KT, and sham group.

## 2. Methods

### 2.1. Design

We designed a prospective, single-blind, randomized, sham-controlled study in which the CT and KT effects were compared.

### 2.2. Approval

The study was approved by the Bioethics Committee of Opole Medical School (Poland: no. KB/01/08/2013) and all subjects provided written informed consent.

### 2.3. Setting, Participants, and Random Allocation of Patients

105 volunteers were recruited from Opole Medical School population to participate. The inclusion criteria were being between 18 and 26 years, being asymptomatic, latent MTrPs in the upper part of the trapezius muscle (pain during examination), the absence of skin allergies, and the consent to physical examination and taping application. The exclusion criteria included any history of upper limb, back or neck severe injury in the last 12 months, surgical intervention, upper limb fractures, neurological diseases or musculoskeletal disorders, pharmacological treatment at present, infection, open wound, rash, decreased blood circulation in the treatment area, a pacemaker, or epilepsy.

Randomization was conducted a priori using the website https://www.random.org/. Participants were randomized into three study groups: cross taping group (CT group), kinesio taping group (KT group), and sham group.

### 2.4. Outcomes and Assessment Procedures

Assessments were collected before and after intervention and after the 24-hour follow-up. The primary outcome was resting bioelectrical activity of UT muscle as assessed by surface electromyography (sEMG) in each group and pain intensity on a visual analog scale (VAS). The secondary outcome was cervical mobility evaluated by tape measurement.

In all patients, a proper tape was applied on the MTrPs of the upper part of the trapezius muscle for three days (72 hours). The evaluation of the MTrPs was conducted while the patient was in relaxed prone position on an examination table, and the upper body was exposed. An experienced physiotherapist was assessing the trapezius muscle bilaterally by palpation with thumb with the same pressure. Four diagnostic criteria for the MTrPs were assumed: a hypersensitive spot in a taut band, pain on spot palpation, restricted ROM, and a referred pain distant to the spot.

**Figure 1:**
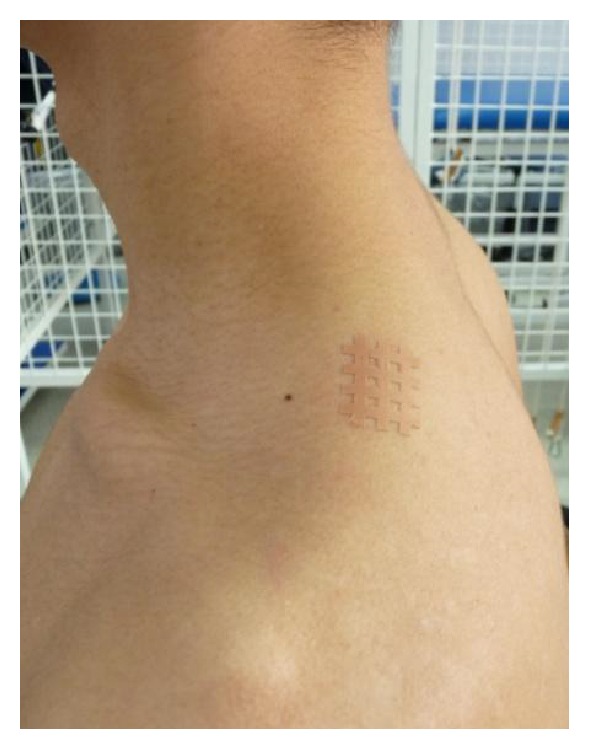
A CT application on MTrPs on the upper part of the trapezius.

**Figure 2:**
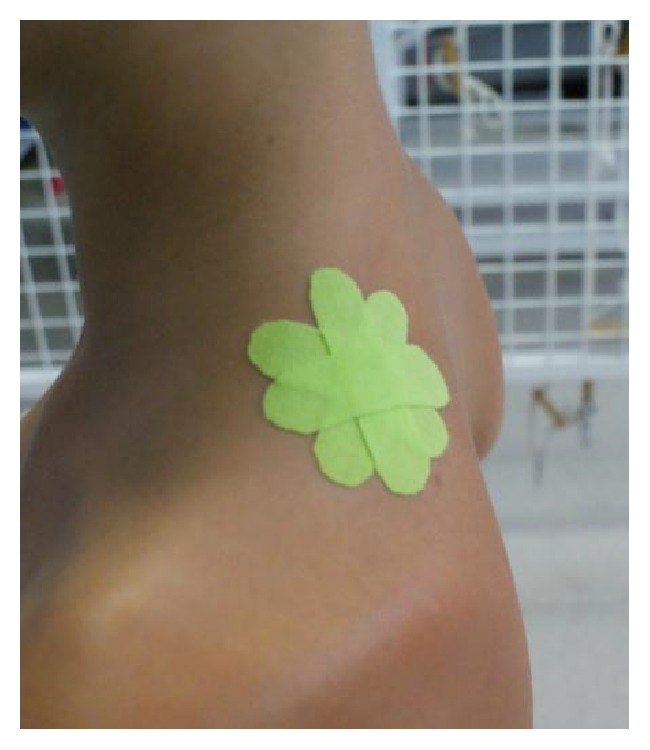
A KT application on MTrPs on the upper part of the trapezius.

**Figure 3:**
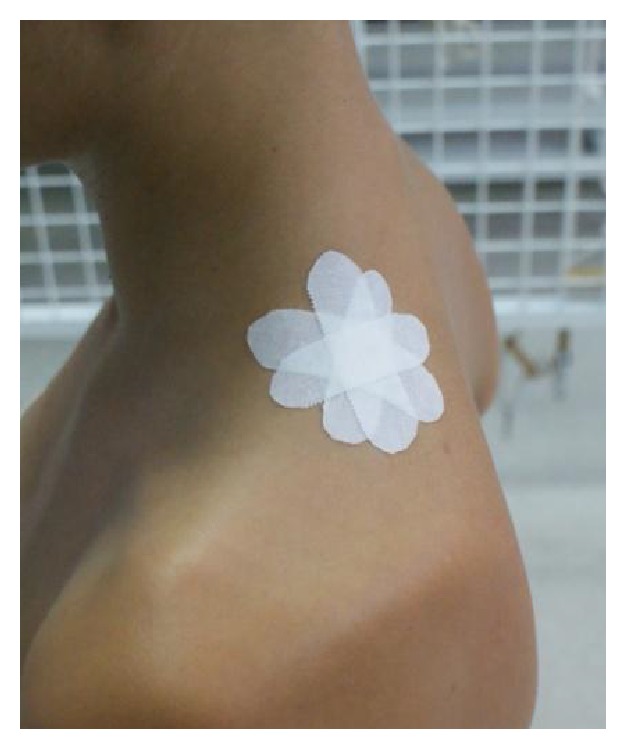
A sham application on MTrPs on the upper part of the trapezius.

MTrPs application with a cross tape (Kumbrink CrossTape, biviax GmbH & Co. KG, 1.5 cm × 2.5 cm, German) was used in CT group (Figure 1). This polyester tape was placed on the upper part of the trapezius, on MTrP spot. In KT group, the Kinesio Tape (Nitto Denko K-Active Tape, 5 m/2.5 cm, Japan) was placed on the same muscle using four “I” strips' application in star shape to create more space directly above an area of pain (space correction) (Figure 2). Each strip was stretched to 50% of available tension [28]. In sham group an adhesive, nonelastic medical tape with no therapeutic influence (Polovis Plus, 5 m/2.5 cm, Poland) was used over the same muscle (four strips in star shape without tension) (Figure 3). Before applications, the skin was shaved, cleaned with alcohol, and dried. All applications were performed by the same researcher (certified KT physiotherapist).

The electromyographic signal was registered by a dual-channel sEMG NeuroTrac ETS device integrated with computer software for digital analysis and report creation (Verity Medical Ltd., United Kingdom). This device is characterized by an amplitude range of 0.2–2000 *μ*V RMS continuous in the frequency band of 2–100 Hz and pulse width from 50 to 450 *μ*S for recording signals generated by muscles. Device sensitivity is established at a level 0.1 *μ*V (4% accuracy; readings +/− 0.3 mV at 200 Hz), with selectable bandpass filter (3 db bandwidth) and 50 Hz notch filter (33 dbs; 0.1% accuracy). The analogue signal recorded by the sEMG electrodes was amplified, filtered, and subsequently transformed into a digital signal. Such signal facilitated statistical analysis of acquired results and allowed for data representation in a graphical form. Mean values of muscle resting bioelectrical activity were given according to root mean square algorithm (RMS). The monopolar, self-adhesive reference electrode was placed on the seventh cervical vertebra.

The electrodes were attached parallel to the muscle fibre orientation over the following muscles: at the UT muscle halfway between the seventh cervical vertebra and the acromion [http://www.seniam.org/].

Pain was recorded by the participant using a 10 cm VAS, where 0 represented no pain and 10 represented unbearable pain. This scale was used to assess the pain during palpation assessment. This assessment was conducted by the same experienced researcher (certified physiotherapist). And during this examination all patients had to evaluate the sensations of pain.

Cervical ROM was measured with a tape measure in centimeters (cm). The range of flexion movement was assessed as the distance from sternal notch to the chin while patients were instructed to bend the head forward; the extension movement was the distance from sternal notch to chin while patients were asked to bend the head backward. Lateral flexion movement was the distance from acromion process to the lowest point of the ear lobe when patients were told to tilt the head to the side opposite the involved UT muscle [44–48].

### 2.5. Statistical Analysis

Data were analysed with the Statistica version 10 for Windows (StatSoft Inc., USA), and the results are presented as the mean ± SD. In order to analyze the changes in bioelectrical activity, VAS score, and ROM between pre-, post-, and follow-up results, the analysis of variance (ANOVA) of Friedman and the Wilcoxon matched-pairs test were used to examine the changes within each group. An independent Kruskal-Wallis ANOVA test and nonparametric multiple comparison test were used for comparison among the three groups. A value of *p* < 0.05 was considered statistically significant.

## 3. Results

**Figure 4:**
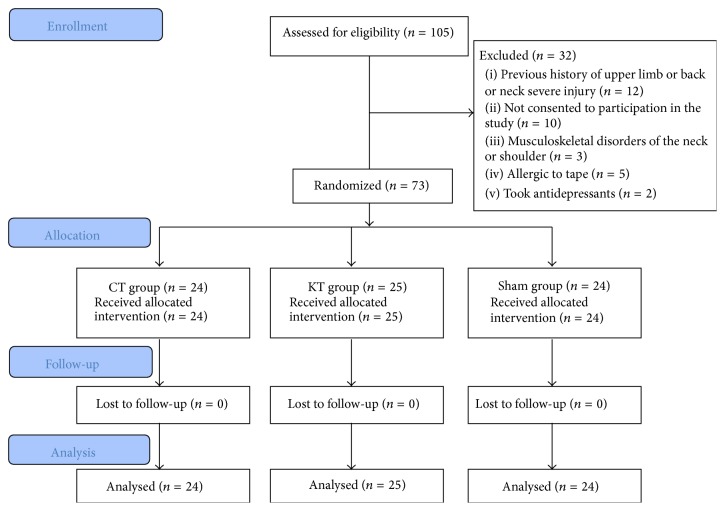
Flow diagram.

A total of 105 people were recruited for this study. 32 were excluded: 12 because they had a previous history of upper limb or back or neck severe injury, 10 because they had not consented to participation in the study, 3 because they had musculoskeletal disorders of the neck or shoulder, 5 because they were allergic to tape, and 2 because they took antidepressants (Figure 4).

The remaining 73 participants were randomized to three groups (CT, KT, and Sham) and all evaluated before, after, and 24 hours after the intervention (follow-up). The demographic characteristics of the participants are presented in Table 1.

In each group, no significant differences were observed in bioelectrical activity between pre-, post-, and follow-up results. There was a significant interaction between results in each group for the VAS score. In fact, in three groups patients had significantly lower pain VAS score after the intervention (main effect in each group: CT—*p* < 0.001, KT—*p* < 0.001, and Sham—*p* < 0.01) (Table 2). Significant differences for the remaining secondary outcomes were detected.

The interaction between pre-, post-, and follow-up results in each group for the range of flexion movement was significant. The range of flexion movement significantly improved in patients after the intervention and after 24 hours (main effect in each group: CT—*p* < 0.0001, KT—*p* < 0.0001, and Sham—*p* < 0.001). Only in KT group, there was a significant interaction between results for the range of lateral flexion movement (*p* < 0.01) (Table 2).

The Kruskal-Wallis ANOVA showed no significant differences in almost all measurements between groups (Table 3). In the KT group, greater decrease of VAS score was found comparatively to sham group (*p* = 0.0018).

## 4. Discussion

This study was conducted to identify CT and KT effect on bioelectrical activity, the level of myofascial pain, and the cervical ROM when taping was applied to subjects with latent MTrPs in their upper part of trapezius muscle. There is scarce number of published studies which show the influence of CT on muscle functions, and there are only a few publications which show the effect of KT in patients with latent MTrPs [8, 11, 36, 38]. The study failed to identify significant effect of KT and CT on muscle bioelectrical activity. Gómez-Soriano et al. [37] found a short-term increase of gastrocnemius muscle activity after KT but it was not maintained up to 24 h. Additionally, the study was performed in healthy subjects, what could diminish the therapeutic effect of taping. Nonetheless, research findings in the literature, concerning the influence of KT on muscle bioelectrical activity, specifically in patients with musculoskeletal disorders [49, 50] report about decreasing effect of Kinesio Tapes on muscle electrical activity. Takasaki et al. [41] investigated whether the activity of the UT muscle varied with the application of tensioned and nontensioned taping. In both cases, applications reduced the UT activity comparing to control group. The inhibitory effect of taping is supported by the findings of Huang et al. [50], who likewise demonstrated that taping over the UT decreased its activity. Lowered electromyographic activity was also noticed by Paoloni et al. [49]. In their research on chronic low back pain patients, KT rapidly reduced abnormal EMG activity of lumbar paraspinal muscles.

According to the study results, only after KT application on UT muscle, the myofascial pain was relieved. However, there was no improvement in range of motion in any of the groups. Bae et al. [8] evaluated the changes in the myofascial pain and ROM of temporomandibular joint when KT was applied to patients with latent MTrPs of the sternocleidomastoid muscle. In this study, they found significant decrease in the VAS score and increase of ROM of temporomandibular joint. Precise mechanism which explains the effect of KT on musculoskeletal pain is not yet fully understood. There are a number of hypotheses indicating a probable analgesic action of KT. The gate control theory seems to be the most fundamental approach, in which the cutaneous stretch stimulation, activated by KT, can interfere nociceptive stimuli reaching the central nervous system and inhibit the pain [11, 49, 51, 52]. Although this theory does not imply to CT, those tapes are unable to provide the stretch to the tissue. The positive effect of KT application on myofascial pain was also observed by García-Muro et al. [38]. In a case report of a patient with shoulder dysfunction caused by the MTrPs, the author showed an objective improvement in the VAS score and the algometry, what was reported as possible consequence of an inactivation of MTrPs in deltoid muscle. Analgesic effects of KT in patients with musculoskeletal dysfunctions are reported most frequently [8, 11, 38, 49, 53].

It is also stated that KT increases blood and lymphatic fluid circulation under the taped area in a consequence of a lifting effect, which creates a wider space between the skin and the muscle [37, 53–55], what may affect muscle functions and result in pain and ROM improvement. With respect to ROM, great importance gains the theory of the influence of KT on fascial tissue [36]. The last decade abounds in scientific exploration concerning the role of fascial system [36, 52]. The direct contact between the fascia and muscular structures suggests that it can take part in transmitting the relative tensioning (evoked by stretched KT tape) to proper receptors and thus elicit the muscle response.

Only in the intragroup comparison we observed significant increase of range of flexion movement in all three groups. However, only in KT group the relevant increase of lateral flexion movement was noted. Those results are compliant with most of the other researches which show the positive effect of KT on ROM [36, 53]. Yoshida and Kahanov [53] reported that KT applied over the lower trunk may increase the ROM of lower trunk flexion as they did not find significant differences for extension and lateral flexion. Both cases, flexion of the lower trunk and lateral flexion of the cervical spine, seem to be the movements where muscle fibers of respective muscle are being stretched the most. Hence, the improvement may be most noticeable in those particular movements. That could explain KT stretch activation of cutaneous and fascial mechanoreceptors resulting in the improvement of muscle excitability. This phenomenon has been also reported in several other studies [38, 53–57].

## 5. Conclusion

The application of all three types of tapes does not influence the resting bioelectrical activity of UT muscle and may not lead to a reduction in muscle tone in the case of MTrPs. However, in comparison to CT and sham, the KT application reduces the subjective pain sensation, what confirms the scientific reports about its analgesic influence. Authors suggest further verification of CT and KT application methods to compare their therapeutic effect and also to compare them with different methods used in the therapy of MTrPs. Therefore, it is appropriate to continue measurements of KT and CT influence on bioelectrical activity of muscles with MTrPs, pain, and cervical ROM. Further experimental research should include a larger number of participants and more objective assessment tools.

## Figures and Tables

**Table 1:** Groups characteristic.

	CT group	KT group	Sham group	*p* value
Number of patients	*n* = 24	*n* = 25	*n* = 24	—

Age [year]				
Range	19.0–23.0	19.0–24.0	19.0–22.0	*p* = 0.2631^*∗*^
Mean	20.2	20.6	19.9	
SD	1.1	1.5	0.8	

Weight [kg]				
Range	43.0–83.0	51.0–87.0	50.0–85.0	*p* = 0.1320^*∗*^
Mean	62.4	66.4	60.6	
SD	10.0	11.6	8.77	

Height [m]				
Range	1.51–1.78	1.57–1.82	1.60–1.75	*p* = 0.2016^*∗*^
Mean	1.68	1.70	1.67	
SD	0.07	0.06	0.04	

BMI [kg/m^2^]				
Range	17.9–28.0	18.6–29.4	18.1–31.2	*p* = 0.4997^*∗*^
Mean	22.0	22.9	21.9	
SD	2.47	3.1	3.2	

Sex				
Female	23	21	24	*p* = 0.0701^*∗∗*^
Male	1	4	0	

Dominant lower limb				
Left	3	1	5	*p* = 0.2008^*∗∗*^
Right	21	24	19	

^*∗*^Kruskal-Wallis test; ^*∗∗*^chi^2^ test.

**Table 2:** Comparison between preintervention, postintervention, and follow-up results in each group.

Outcomes	Group	Measurement			*p* value
		Preintervention	Postintervention	Follow-up	(main effect of Friedman ANOVA)
Resting bioelectrical activity (*µ*V) Mean ± SD	CT	6.8 ± 4.8	4.5 ± 2.7	4.2 ± 2.6	*p* = 0.1152
	KT	6.2 ± 4.3	5.0 ± 3.3	4.6 ± 2.8	*p* = 0.3260
	Sham	7.4 ± 7.6	4.7 ± 2.5	3.9 ± 1.9	*p* = 0.0542

Visual analogue scale (VAS) Mean ± SD	CT	7.2 ± 1.2^*∗∗*^	5.8 ± 1.6^*∗*^	5.1 ± 1.8^*∗∗∗*^	**p** = 0.0001
	KT	6.8 ± 1.8^*∗∗*^	4.0 ± 2.0^*∗*^	5.2 ± 2.4^*∗∗∗*^	**p** = 0.0001
	Sham	6.4 ± 1.6^*∗∗*^	5.7 ± 2.0	4.9 ± 2.2^*∗∗∗*^	**p** = 0.0011

The range of flexion movement (cm) Mean ± SD	CT	2.6 ± 0.7^*∗∗*^	2.0 ± 0.5^*∗*^	1.4 ± 0.9^*∗∗∗*^	**p** = 0.0000
	KT	3.1 ± 1.0^*∗∗*^	2.2 ± 1.0^*∗*^	1.7 ± 1.1^*∗∗∗*^	**p** = 0.0000
	Sham	3.1 ± 0.8^*∗∗*^	2.7 ± 0.9^*∗*^	2.4 ± 0.7^*∗∗∗*^	**p** = 0.0004

The range of extension movement (cm) Mean ± SD	CT	8.0 ± 1.2	8.0 ± 1.6	8.0 ± 1.1	*p* = 0.8140
	KT	7.8 ± 1.6	7.9 ± 1.5	8.6 ± 1.6	*p* = 0.3068
	Sham	8.1 ± 1.3	7.6 ± 1.0	8.6 ± 1.2	*p* = 0.3068

The range of left lateral flexion movement (cm) Mean ± SD	CT	5.3 ± 1.3	5.2 ± 1.0	5.9 ± 0.9	*p* = 0.0056
	KT	5.4 ± 1.2^*∗∗*^	6.0 ± 0.9^*∗*^	6.0 ± 0.8	**p** = 0.0010
	Sham	5.3 ± 1.0	5.5 ± 0.7	5.9 ± 0.7	*p* = 0.0057

The range of right lateral flexion movement (cm) Mean ± SD	CT	5.2 ± 1.1	5.4 ± 1.2	5.7 ± 1.0	*p* = 0.0705
	KT	5.3 ± 1.1	5.8 ± 1.0	5.8 ± 0.9	*p* = 0.0314
	Sham	5.4 ± 0.9	5.4 ± 0.7	6.0 ± 0.6	*p* = 0.0051

Post hoc analysis:

^*∗*^Statistically significant comparison between pre- and postresults (*p* < 0.05).

^*∗∗*^Statistically significant comparison between pre- and follow-up results (*p* < 0.05).

^*∗∗∗*^Statistically significant comparison between post- and follow-up results (*p* < 0.05).

**Table 3:** Comparison of the results between CT, KT, and sham groups.

Outcomes	Results^*∗*^	Differences between groups			*p* value (main effect of Kruskal-Wallis ANOVA)
		(post hoc analysis)			
		CT/KT	CT/sham	KT/sham	
Resting bioelectrical activity (*µ*V) Mean ± SD	I	*p* > 0.05	*p* > 0.05	*p* > 0.05	*p* = 0.5892
	II	*p* > 0.05	*p* > 0.05	*p* > 0.05	*p* = 0.7014
	III	*p* > 0.05	*p* > 0.05	*p* > 0.05	*p* = 0.3939

Visual analogue scale (VAS) Mean ± SD	I	*p* > 0.05	*p* > 0.05	**p** = 0.0018	**p** = 0.0017
	II	*p* > 0.05	*p* > 0.05	*p* > 0.05	*p* = 0.6259
	III	*p* > 0.05	*p* > 0.05	*p* > 0.05	*p* = 0.1897

The range of flexion movement (cm) Mean ± SD	I	*p* > 0.05	*p* > 0.05	*p* > 0.05	*p* = 0.3859
	II	*p* > 0.05	*p* > 0.05	*p* > 0.05	*p* = 0.1440
	III	*p* > 0.05	*p* > 0.05	*p* > 0.05	*p* = 0.4227

The range of extension movement (cm) Mean ± SD	I	*p* > 0.05	*p* > 0.05	*p* > 0.05	*p* = 0.4890
	II	*p* > 0.05	*p* > 0.05	*p* > 0.05	*p* = 0.2646
	III	*p* > 0.05	*p* > 0.05	*p* > 0.05	*p* = 0.0529

The range of left lateral flexion movement (cm) Mean ± SD	I	*p* > 0.05	*p* > 0.05	*p* > 0.05	*p* = 0.1427
	II	*p* > 0.05	*p* > 0.05	*p* > 0.05	*p* = 0.9505
	III	*p* > 0.05	*p* > 0.05	*p* > 0.05	*p* = 0.0541

The range of right lateral flexion movement (cm) Mean ± SD	I	*p* > 0.05	*p* > 0.05	*p* > 0.05	*p* = 0.2622
	II	*p* > 0.05	*p* > 0.05	*p* > 0.05	*p* = 0.8262
	III	*p* > 0.05	*p* > 0.05	*p* > 0.05	*p* = 0.0862

^*∗*^Results:

Result I—post- minus pre-intervention results.

Result II—follow-up minus pre-intervention results.

Result III—follow-up minus post-intervention results.
